# GlaR (YugA)—a novel RpiR‐family transcription activator of the Leloir pathway of galactose utilization in *Lactococcus lactis* IL1403

**DOI:** 10.1002/mbo3.714

**Published:** 2018-08-11

**Authors:** Tamara Aleksandrzak‐Piekarczyk, Katarzyna Szatraj, Katarzyna Kosiorek

**Affiliations:** ^1^ Institute of Biochemistry and Biophysics Polish Academy of Sciences (IBB PAS) Warsaw Poland

**Keywords:** galactose assimilation, *Lactococcus lactis*, Leloir pathway, sugar metabolism, transcription regulation

## Abstract

Bacteria can utilize diverse sugars as carbon and energy source, but the regulatory mechanisms directing the choice of the preferred substrate are often poorly understood. Here, we analyzed the role of the YugA protein (now designated GlaR—Galactose–lactose operon Regulatory protein) of the RpiR family as a transcriptional activator of galactose (*gal* genes) and lactose (*lac* genes) utilization genes in *Lactococcus lactis* IL1403. In this bacterium, *gal* genes forming the Leloir operon are combined with *lac* genes in a single so‐called *gal–lac* operon. The first gene of this operon is the *lacS* gene encoding galactose permease. The *glaR* gene encoding GlaR lies directly upstream of the *gal–lac* gene cluster and is transcribed in the same direction. This genetic layout and the presence of *glaR* homologues in the closest neighborhood to the Leloir or *gal–lac* operons are highly conserved only among *Lactococcus* species. Deletion of *glaR* disabled galactose utilization and abrogated or decreased expression of the *gal–lac* genes. The GlaR‐dependent regulation of the *gal–lac* operon depends on its specific binding to a DNA region upstream of the *lacS* gene activating *lacS* expression and increasing the expression of the operon genes localized downstream. Notably, expression of *lacS*‐downstream genes, namely *galMKTE*,* thgA* and *lacZ*, is partially independent of the GlaR‐driven activation likely due to the presence of additional promoters. The *glaR* transcription itself is not subject to catabolite control protein A (CcpA) carbon catabolite repression (CRR) and is induced by galactose. Up to date, no similar mechanism has been reported in other lactic acid bacteria species. These results reveal a novel regulatory protein and shed new light on the regulation of carbohydrate catabolism in *L. lactis* IL1403, and by similarity, probably also in other lactococci.

## 1 INTRODUCTION

Lactose, a disaccharide comprised of galactose linked through a β‐glycosidic bond to the C_4_ of glucose, is the dominant sugar found in milk. Lactic acid bacteria (LAB) are capable of growth in milk owing to an efficient use of lactose as a carbon source. Because of the high efficiency and economic relevance of lactose fermentation, numerous studies have focused on LAB. Lactose utilization genes have been characterized in many LAB species, and it has been shown that they can take up lactose by two principally different ways including the lactose‐specific phosphotransferase system (*lac*‐PTS) and secondary transporters such as lactose‐galactose antiporters and lactose‐H^+^ symporters (reviewed by Aleksandrzak‐Piekarczyk, 2013). The secondary transport systems transfer unphosphorylated lactose via specific permeases of the LacS subfamily (TC No. 2.A.2.2.3) belonging to the 2.A.2 glycoside‐pentoside‐hexuronide (GPH) family (Saier, 2000). After its import, lactose is hydrolyzed by β‐galactosidase to glucose and galactose. Then, glucose is further metabolized via glycolysis, while the galactose moiety can be either released into the medium or converted into glucose‐1‐phosphate (Glc‐1‐P), which enters glycolysis following conversion to Glc‐6‐P. The conversion into Glc‐1‐P is performed by the action of four enzymes that constitute the Leloir pathway (De Vos, 1996; Poolman, 1993; Vaughan, van den Bogaard, Catzeddu, Kuipers, & de Vos, 2001). This pathway, discovered by L. F. Leloir and coworkers in 1950s (reviewed in Frey^1996^), consists of the crucial enzyme galactokinase (GalK) plus hexose‐1‐P uridylyltransferase (GalT) and UDP‐glucose 4‐epimerase (GalE) that perform the conversion of galactose into glucose‐1‐P. Found more recently, an additional enzyme, the GalM mutarotase (aldose‐1‐epimerase), is involved in the interconversion of the galactose α‐ and β‐anomers (Bouffard, Rudd, & Adhya, 1994).

In LAB, the Leloir pathway genes may be present on their own (*gal* genes) or combined with genes for lactose metabolism (*lac* genes) (Grossiord, Vaughan, Luesink, & de Vos, 1998; Vaillancourt, Moineau, Frenette, Lessard, & Vadeboncoeur, 2002). In the latter case, in addition to the *galKTEM* genes (depending on a LAB species, in a variable genomic organization and order), extra genes such as *lacZ* (β‐galactosidase) and *lacA* (*thgA*; thiogalactoside acetyltransferase) genes are present (*gal–lac* operon) (Aleksandrzak‐Piekarczyk, Kok, Renault, & Bardowski, 2005; Poolman, Royer, Mainzer, & Schmidt, 1990; Vaillancourt et al., 2002). Directly upstream of these genes encoding enzymes catalyzing lactose hydrolysis and/or galactose conversion, or within this operon, a gene encoding specific permease for lactose or galactose uptake may also be present (Grossiord et al., 1998; Vaillancourt et al., 2002).

The uptake and metabolism of sugars is mastered by numerous regulatory proteins which form a regulatory network detecting environments and setting the catabolic abilities of the cell, thus helping to maintain energy efficiency. Based on their specificity, two groups of regulators are distinguished, general and secondary ones (Guédon, Jamet, & Renault, 2002; Mayo et al., 2010). In most low‐GC gram‐positive bacteria, the main general regulator is catabolite control protein A (CcpA) (Hueck & Hillen, 1995), which acts by binding to 14‐nucleotide DNA target sites known as *cre* (catabolite responsive elements), conducting carbon catabolite activation (CCA) or repression (CCR) (Weickert & Chambliss, 1990). The *cre* sites are found in promoter regions of the CCR‐ and CCA‐sensitive genes and the binding by CcpA to them is strongly stimulated by Ser46‐phosphorylated HPr protein (Deutscher, 2008). In *Lactococcus lactis* strains, CcpA has been shown to repress transcription of different genes associated with the uptake of β‐glucosides, fructose, galactose, and lactose and to activate the glycolytic operon *las* (Aleksandrzak‐Piekarczyk, Polak, Jezierska, Renault, & Bardowski, 2011; Aleksandrzak‐Piekarczyk et al., 2005; Barrière et al., 2005; Luesink, van Herpen, Grossiord, Kuipers, & de Vos, 1998; Monedero, Kuipers, Jamet, & Deutscher, 2001). Sugar catabolism can also be mastered by specific secondary regulators, common in LAB and acting locally, falling to diverse protein families such as LacI, LysR, AraC, GntR, DeoR, RpiR, or BglG. In lactococci, regulators belonging to some of these families have been shown to positively or negatively control genes directing utilization of sugars such as α‐galactosides, β‐glucosides, fructose, lactose, maltose, sucrose, and xylose (Aleksandrzak‐Piekarczyk, Stasiak‐Różańska, Cieśla, & Bardowski, 2015; Andersson & Rådström, 2002; Bardowski, Ehrlich, & Chopin, 1994; Barrière et al., 2005; Boucher, Vadeboncoeur, & Moineau, 2003; Erlandson et al., 2000; Rauch & de Vos, 1992; Van Rooijen & de Vos, 1990).

The mechanisms of transcriptional regulation of the Leloir pathway genes have been elucidated in some LAB species. *Gal–lac* operons are frequently regulated by specific transcription regulators, which belong to the LacI type. In *Streptococcus* (*S*.) *thermophilus* and *S. mutans,* GalR acts as a transcription activator and repressor of the *lac* and *gal* operons, respectively (Ajdić & Ferretti, 1998; Vaughan et al., 2001). In both species, the GalR‐encoding *galR* gene is oriented divergently from the structural genes of the Leloir operon. In *Lactobacillus casei*, a potential transcription regulatory gene, *galR*, has been identified in the *gal* operon and is transcribed in the same direction (Bettenbrock & Alpert, 1998). In *Lactobacillus helveticus*, the inducible genes *lacLM* (encoding β‐galactosidase) of the unusually organized *gal* and *lac* gene cluster are regulated at the transcriptional level by LacR repressor (Fortina, Ricci, Mora, Guglielmetti, & Manachini, 2003). No specific regulatory genes have been identified for the Leloir operon in *L. lactis* to date, albeit it has been demonstrated that expression of *gal* genes is under CcpA‐dependent catabolic repression (Luesink et al., 1998; Zomer, Buist, Larsen, Kok, & Kuipers, 2007).

We propose that YugA activates expression of *lacS* and the *lac–gal* genes localized downstream by binding to the *lacS* upstream DNA region containing a putative promoter. Because of this newly identified regulatory function of YugA, we propose to re‐name it GlaR (galactose–lactose operon Regulatory protein). To the best of our knowledge, this is the first report exploring a specific GlaR‐dependent regulatory mechanism of the Leloir pathway genes in *L. lactis* IL1403 at the molecular level. We examined the effects of *glaR* deletion and found that the lack of GlaR precludes the strain's growth in galactose‐containing media and abolishes *lacS* gene expression. These results shed new light on the regulation of carbohydrate catabolism in this biotechnologically important bacterium and reveal a new regulatory protein. Notably, the described mechanism of control of galactose and lactose catabolism by enzymes of the Leloir utilization pathway is unique among LAB.

## 2 MATERIALS AND METHODS

### 2.1 Bacterial strains, media, and plasmids

Bacterial strains and plasmids used in this study are shown in Table 1. *Escherichia coli* cells were cultivated in Luria–Bertani (LB) medium (Wood, 1983) at 37°C, and *L*. *lactis* was grown in M17 medium (Terzaghi & Sandine, 1975) or in CDM (Sissler et al., 1999). M17 and CMD were supplemented with 1% glucose (G‐M17 or G‐CDM), or 1% cellobiose (C‐M17 or C‐CDM), or 1% galactose (Gal‐M17 or Gal‐CDM), or 1% galactose with 1% cellobiose (GalC‐M17 or GalC‐CDM). When necessary, ampicillin (Amp; 100 μg ml^−1^ for *E*. *coli*) or erythromycin (Em; 100 μg ml^−1^ for *E*. *coli* and 5 μg ml^−1^ for *L*. *lactis*) was added to the medium. Solidified media contained 1.5% agar and, when required for *E. coli*, 1 mM IPTG (isopropyl β‐D‐thiogalactopyranoside) and 50 μg ml^−1^ for X‐gal (5‐bromo‐4‐chloro‐3‐indolyl‐β‐D‐galactopyranoside).

**Table 1:** Bacterial strains, plasmids, and primers

Strain, plasmid, or primer pair	Relevant genotypic or phenotypic properties^a^	Source and/or reference^b^
Strains		
*L. lactis*		
IL1403	Gal^+^, plasmid‐free wild‐type, host strain	INRA (Chopin et al.^1984^)
LL302	*L. lactis* MG1363 derivative, RepA^+^	(Leenhouts et al.^1998^)
IL1403Δ*glaR*	Gal^‐^, Δ*glaR*, Em^s^ *,* plasmid‐free, IL1403 derivative	This study
IL1403*ccpA* ^‐^	Lac^+^ *,* CcpA^‐^ (IS*S1*), Em^s^, plasmid‐free, IL1403 derivative	(Aleksandrzak‐Piekarczyk et al., 2005)
IL1403Δ*glaR*‐pGhost9*glaR*	Gal^+^, Em^r^, IL1403Δ*glaR* derivative carrying pGhost9*glaR*	This study
*E. coli*		
TG1	Δ(*hsdMS‐mcrB*)5 Δ(*lac‐proAB*) *supE thi‐1* F’(*traD36 proAB* ^*+*^ *lacI* ^q^ *Z*Δ*M15*)	(Gibson^1984^)
EC1000	Km^r^, RepA^+^ MC1000	(Leenhouts et al.^1996^)
BL21	B F^‐^ *ompT dcm lon hsdSB*(rB^‐^ mB^‐^) gal [malB+]K‐12(λS)	(Miroux and Walker^1996^)
Plasmids		
pGEM‐T	Amp^r^, M13*ori*, linear T‐overhang vector	Promega
pGhost9	Em^r^, *repA* (Ts)	INRA (Maguin et al.^1996^)
pIL253	Em^r^, high‐copy number lactococcal vector	(Simon and Chopin^1988^)
ptXB1	Amp^r^, M13*ori*,* rop*,* lacI*, Mxe GyrA intein	New England Biolabs
Recombinant plasmids		
pGhost9Δ*glaR*	Em^r^, pGhost9 carrying *glaR* upstream, and downstream regions	This study
pGhost9*glaR*	Em^r^ *,* pGhost9 carrying *glaR* under the control of its promoter	This study
Primers^c^		
For deletion and complementation of the *glaR* gene		
*glaR*UPf/*glaR*UPr	CC**ATCGAT**TCAAGTTCCCAAACGCTCC/GGA**GAATTC**GCCAAGTATAGGATTCAGC	
*glaR*DOWNf/*glaR*DOWNr	GGA**GAATTC**CAAGAGTAGTCTTGAGGTG/AAGATGACATAATCCCACCAACAAC	
*glaR*for/*glaR*rev	GCTAAGACCGCAGCTTC/GACCAGAAGGCAATGTC	
ptXB1for/*glaR*BamHrev	GTGAGCGGATAACAATTCC/GGATCCTTATTGTTTTAAAGTATAAATGG	
For qPCR amplifications		
LlGlaRaF/LlGlaRaR	TGCAACTTTTCCGTAAGCCC/TTGGGATTTTGTCCTTTGGC	
LlLacSaF/LlLacSaR	CTGGAACACCACATGAGGATGC/AAGATGACATAATCCCACCAACAAC	
LlGalMaF/LlGalMaR	TGACCATCCTTTCTTGTTAGACCAG/CCATGGTGCACTTGCTTTTTC	
LlGalKaF/LlGalKaR	AACAAGCCGGTGTCTTGGG/TCCAACTTTGTTGAACCAGAACC	
LlGalTaF/LlGalTaR	AAAAAGACCCCAAAGCCATTG/ATTGGAAGCCCCAGTCTTCG	
LlThgAaF/LlThgAaR	CCAAATGTTACGATTGACACGG/AGACTCCCTGCGCCAATCAC	
LlLacZaF/LlLacZaR	GAAAGCACTTCTTGTTCGTGGAG/TCACACAATTCATACCAGCGTG	
LlGalEaF/LlGalEaR	GCCTGATGGAACTTGTATTCGTG/CCTGTTACTTTTCGTGCGGTTTC	
LlYufCaF/LlYufCaR	TTGCAGGAGAAACTTTGACGG/TCTGCCCACGGAATAGCAC	
LlPurMaF/LlPurMaR	ATTGCGTAGCCATGTGCGTC/CTGTTTCTCCACCAATCAGCG	
LlTufaF/LlTufaR	CGTGACCTCTTGAGCGAATACG/GAGTGGTTTGTCAGTGTCGCG	
For amplification of nucleotides for EMSA		
*glaR*for/*glaR*rev	GCCAGAGTCCTAATGAAAG/CATGGCTTACTATGCCC	
*lacS*for/*lacS*rev	CTAATTGATGCTTACTCC/CTTTCATGGGAATCCTCC	
*galM*for/*galM*rev	GCCTATCCTGGTGCAAC/CCATGATATTTCCTAACT	
*galT*for/*galT*rev	GTTGTCGGTTATCCAGC/CAAGTGGCTCAATCGTTCC	
*thgA*for/*thgA*rev	CAGGAAGCAGTTGGAGAAG/CAGCCAGAGCAACAAATGG	
*galE*for/*galE*rev	GGACATTGGCATCTACTTG/CTGCCACATCGTAACCACG	
*yufA*for/*yufA*rev	CTTGAAGTGCTTGAAACC/CCATTACATTTTCATGACG	

^a^Amp, ampillicin; Em, erythromycin; Km, kanamycin; r, resistance; s, sensitivityl; CcpA, catabolite control protein A^b^INRA, Institut National de la Recherche Agronomique (Jouy‐en‐Josas, France).^c^All primers were designed on the basis of the *L. lactis* IL1403 genome nucleotide sequence, NCBI with accession no. AE005176 (http://www.ncbi.nlm.nih.gov/genome). To certain primers, restriction sites were added for digestion with **EcoRI** or **ClaI.**

### 2.2 Construction of *glaR* deletion mutant and complementing plasmid

*Lactococcus lactis* IL1403 *glaR* deletion strain (*L. lactis* IL1403Δ*glaR*) was generated by double crossover between pGhost9 carrying DNA fragments flanking the *glaR* gene and the corresponding chromosomal region. The *glaR* upstream and downstream DNA fragments were amplified with, respectively, the *glaR*UPf/*glaR*UPr and *glaR*DOWNf/*glaR*DOWNr primer pairs (Table 1). The obtained DNA fragments were cloned in the proper orientation in the integrative vector pGhost9, producing pGhost9Δ*glaR*. This deletion plasmid was transported into *L. lactis* IL1403 and homologous recombination was enforced by 10^−3^ dilution of an overnight culture and incubation at nonpermissive temperature (38°C). Cells harboring pGhost9Δ*glaR* in the chromosome were cultivated at 38°C on G‐M17Em. Removal from the chromosome and elimination of pGhost9 from *L. lactis* were performed by growing the integrants in G‐M17 without antibiotic for at least 100 generations at the permissive temperature (28°C). The genomic organization of the resulting *glaR* deletion strain (*L. lactis* IL1403Δ*glaR*) was confirmed by determining its sensitivity to Em and by sequencing of the mutated region.

To complement the *glaR* deletion, the *glaR* gene containing its putative promoter region was amplified using *glaR*UPf and *glaR*DOWNr primers (Table 1) and ExTaq polymerase giving the *glaR*(A) insert. The insert was introduced by TA cloning into pGhost9, as described by Radziwill‐Bienkowska et al. (2016). Shortly, pGhost9 was blunt‐linearized with EcoRV and, to add 3′ thymidine overhangs, treated with terminal deoxynucleotidyl transferase (TdT; Thermo Fisher Scientific, USA) and 2′,3′‐dideoxythymidine‐5′‐triphosphate (ddTTP; Affymetrix, USA). The obtained pGhost9(T) was ligated with the *glaR*(A) insert and cloned in *E. coli* EC1000. The pGhost9*glaR* plasmid was isolated, verified by sequencing of the *glaR* insert, and transformed into *L. lactis* IL1403Δ*glaR* to give *L. lactis* IL1403Δ*glaR*‐pGhost9*glaR*.

### 2.3 Quantification of gene expression by reverse transcription‐quantitative PCR (RT‐qPCR)

RNA was isolated following manufacturer's instructions with the use of GeneMATRIX Universal RNA Purification Kit (EURx, Poland) from 10 ml of *L. lactis* IL1403 and *L. lactis* IL1403Δ*glaR* cultures grown in G‐M17, C‐M17, Gal‐M17 or GalC‐M17 and collected from midexponential phase (OD_600 _= 0.6). RNA was isolated from at least three independent cultures.

First‐strand cDNA was obtained from DNAse I (Sigma‐Aldrich, USA)‐treated RNA with random primers by the use of the RevertAid(TM) First‐Strand cDNA Synthesis Kit (Thermo Fisher Scientific) according to manufacturer's instructions. qPCR assays on the cDNA were carried out in a 7500 Real‐Time PCR System (Applied Biosystems, USA) and following the previously described methodology (Aleksandrzak‐Piekarczyk et al., 2015). Specific primers for genes (Table 1) were created with Primer Express software (Applied Biosystems). The results were normalized to the *L. lactis* IL1403 reference genes *tuf* and *purM* coding for elongation factor TU and phosphoribosylaminoimidazole synthetase, respectively.

### 2.4 Growth testing for carbon source utilization

Growth tests were performed using a Microbiology Reader Analyser, Bioscreen C (Oy Growth Curves Ab Ltd, Finland) in 200 μl of CDM with the required sugars (glucose, galactose or cellobiose). OD_600_ of the bacterial cultures was recorded every 60 mins of growth up to 40 hr at 30°C. The assays were carried out in triplicate.

### 2.5 Overproduction and purification of GlaR

The self‐cleavable IMPACT^TM^ affinity tag system (New England Biolabs, USA) was used to purify the GlaR protein. *E. coli* BL21 competent cells were transformed with the pTXB1 plasmid carrying the *glaR* gene. The obtained transformants were verified by colony PCR, with specific primers ptXB1for and *glaR*BamHrev. LB medium (600 ml) containing 100 μg/ml ampicillin was inoculated with a freshly grown colony and incubated at 37°C with shaking until an OD_660_ of 0.5 was reached. After induction of the *glaR* gene expression using 0.3 mM IPTG, the culture was incubated overnight at 18°C. Then, the cells were pelleted by centrifugation (3000xg, 10 min, 4°C) and stored at −20°C until use. All subsequent purification steps were carried out at 4°C. The frozen cells were resuspended in 10 ml of column buffer A (25 mM Tris‐HCl, pH 8.0; 500 mM NaCl; 10% glycerol) and were disrupted by High Pressure Homogenizer Emulsiflex (Avestin Inc., Canada). After centrifugation (15,000×*g*, 30 min, 4°C), 5 ml of the clear supernatant was loaded (at 0.5–1 ml/min) onto a polypropylene column (Qiagen, Germany) with 2 ml of chitin beads (New England Biolabs) previously equilibrated with 20 ml of chitin column buffer A. Next, the resin was washed with 20 ml of the same buffer and then with 3 ml of the cleavage buffer B (25 mM Tris‐HCl, pH 8.0; 100 mM KCl; 50 mM DTT; 1 mM MgCl_2_; 10% glycerol). The GlaR protein was released from the chitin beads after 16 hrs of incubation at 23°C. The eluted GlaR protein, with no extra residues, was concentrated using an Amicon filter device (Millipore, USA) and analyzed on 15% SDS‐polyacrylamide gel with Coomassie staining.

The protein band was cut out from the gel, reduced with 100 mM DTT (30 min, 56°C), alkylated at darkroom with 0.5 M iodoacetamide (45 min, RT), and digested overnight with trypsin (37°C 10 ng μl^−1^; Promega, USA). Peptide mixture was concentrated, desalted on a RP‐C18 precolumn (Waters, USA), and separated on a nano‐Ultra Performance Liquid Chromatography (UPLC) RP‐C18 column (BEH130 C18; Waters), using a 160‐min gradient from 5% to 30% of acetonitrile. Measurements were taken with the Orbitrap Velos spectrophotometer (Thermo Fisher Scientific), working in the regime of data‐dependent MS to MS/MS switch with HCD type peptide fragmentation. Identification of proteins was performed using the Mascot search engine with the probability‐based algorithm. Data were searched with automatic decoy database and filtered to obtain a false discovery rate below 1%.

Protein concentration was determined using Bradford assay on a NanoDrop spectrophotometer (Thermo Scientific).

### 2.6 Electrophoretic mobility‐shift assay (EMSA)

EMSA was performed using 1 nM of double‐stranded DNA fragments (~300 bp) generated by PCR with specific primer pairs (Table 1) multiplying the upstream DNA regions of selected genes (*glaR, lacS, galM, galT, thgA,* and *galE*). The PCR product of the *yufA* upstream DNA region served as a negative control. The DNA fragments were incubated with increasing quantities of the GlaR protein (0; 1; 2; 2.5; 3; 3.5; 4; 4.5; 5, and 6 μM) in 10× binding buffer (10 mM Tris‐HCl, pH 8.5; 10 mM MgCl_2_; 100 mM KCl; 0.1 mg/ml BSA), supplemented with 250 mM galactose in a total volume of 20 μl. After 20 mins of incubation at 37°C, the samples were separated on 5% polyacrylamide gel in 0.5× Tris‐borate‐EDTA buffer (TBE). The amount of GlaR protein giving best results was evaluated as 4 μM. The final EMSA for all upstream DNA sequences of selected genes was performed in previously described conditions, using the optimized amount of the protein (Figure 4).

## 3 RESULTS

### 3.1 Structural characterization of DNA region following the *glaR* gene

**Figure 1:**
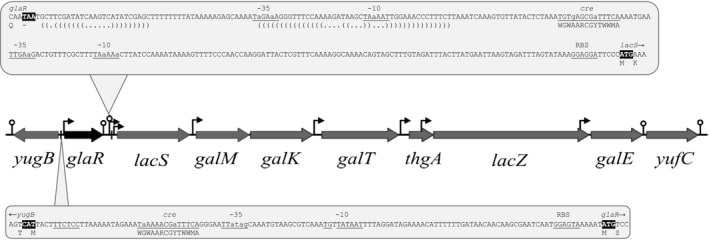
Organization of the *gal–lac* operon with surrounding genes in *L. lactis* IL1403. Lollipops and brackets indicate potential transcription terminators. Bent arrows and underlined nucleotides indicate potential promoter regions. White‐on‐black font marks START and STOP codons. RBS indicates ribosome‐binding site. Vertical lines and underlined *cre* indicate catabolite responsive element with *cre* consensus sequence (WGWAARCGYTWWMA). The picture presents the correct sequence of *lacS*, which at the NCBI database is annotated as a pseudogene due to the deletion of an adenine 446. We confirmed correctness of *lacS* by its sequencing

Figure 1 illustrates the chromosomal region of the *glaR* gene (formerly denoted *yugA*) in *L. lactis* IL1403. The product of *glaR*, the GlaR protein, is highly similar with other transcription regulators of the RpiR family as it is a two‐domain protein and comprises a 59‐residue N‐terminal DNA‐binding helix‐turn‐helix (HTH) domain and a 99‐residue sugar isomerase (SIS) motif at its C‐terminus (http://pfam.sanger.ac.uk/). The YugA amino acid sequence is 100% identical with its orthologues encoded in *L. lactis* subsp. *lactis* genomes, 98% with *L. lactis* subsp. *cremoris* and only 70% with *Lactococcus garvieae*. The *glaR* gene is preceded by a putative promoter, of which the ‐10 region is in full agreement with the promoter consensus sequence, defined as TATAAT (Browning & Busby, 2004), and a potential CcpA‐binding *cre* site with two mismatches with the *cre* consensus, in *L. lactis* defined as WGWAARCGYTWWMA (Zomer et al., 2007) (Figure 1). The *glaR* gene is followed by a potential rho‐independent terminator (Figure 1) with a free energy value (ΔG) of −15 kcal/mol. The presence of these transcriptional signals indicates that *glaR* may form a single‐gene operon regulated by the CcpA protein.

Located downstream of *glaR* and transcribed in the same direction are the genes of Leloir pathway cluster, which in *L. lactis* IL1403 in addition to the four galactose genes contains a sugar permease gene (*lacS*) and predicted genes for lactose assimilation such as *lacZ* (β‐galactosidase) and *thgA* (thiogalactoside acetyltransferase). An in silico analysis identified several putative promoters preceding the *lacS*,* galMKT*,* thgA, lacZ*, and *galE* genes (Figure 1) suggesting multiple transcription start sites within the operon. Two of the identified potential −10 regions, those upstream of *lacS* and *galE*, are in full agreement with the promoter consensus sequence, whereas none of the promoters found contains a sequence identical to the −35 consensus TTGACA (Browning & Busby, 2004). Downstream of the *galE* gene, a potential rho‐independent terminator with a ΔG value of −11.4 kcal/mol, was identified.

### 3.2 The genetic organization of *glaR* followed by the Leloir operon is highly conserved among lactococci

Among the 39 fully sequenced *Lactococcus* spp. genomes deposited in the GenBank database (as of January, 2018), *glaR* homologues were identified in 36 strains of *L. lactis* and *L. garvieae*, but were absent from *Lactococcus piscium* and *Lactococcus raffinolactis*. In all the cases, *glaR* lies directly upstream of the Leloir operon and is transcribed in the same direction. Further comparative analyses revealed that this genetic layout is specific for lactococci only, as in other species of the order *Lactobacillales*, in some of which more distant *glaR* homologues are present (over 30% amino acid sequence identity), this gene is never adjacent to the Leloir cluster.

### 3.3 GlaR is crucial for *L. lactis* IL1403 growth on galactose

**Figure 2:**
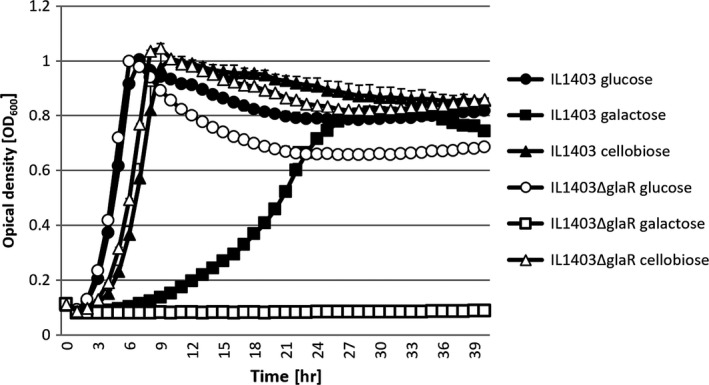
Kinetics of *L. lactis* IL1403 wild‐type and Δ*glaR* strains in CDM supplemented with different sugars

To assess the possible role of GlaR, a *L. lactis* IL1403Δ*glaR* mutant strain was constructed lacking the *glaR* gene and its growth was tested in CDM with different sugars and compared with its parental wild‐type IL1403 strain. No significant differences were found between the growth of these two strains in G‐CDM or C‐CDM, but in a galactose‐supplemented medium, the mutant lacking GlaR was unable to grow completely (Figure 2).

Transformation of pGhost9*glaR* into IL1403Δ*glaR* that led to the creation of the *L. lactis* IL1403Δ*glaR*pGhost9*glaR* strain, fully reversed the effect of the *glaR* deletion, restoring the mutant's growth in medium supplemented with galactose.

### 3.4 GlaR is a transcriptional activator of the *gal–lac* genes

**Figure 3:**
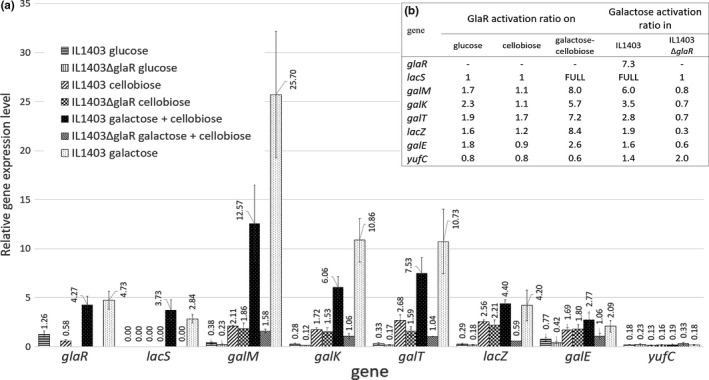
The relative gene expression levels in *L. lactis* IL1403 wild‐type and IL1403Δ*glaR*. (A) mRNA levels determined by RT‐qPCR in relation to *tuf* and *purM*. (B) GlaR and galactose activation ratios calculated, respectively, as a quotient of relative gene expression in IL1403 and IL1403Δ*glaR*, and as a quotient of relative gene expression in strains grown in GalC‐M17 and C‐M17. “FULL” indicates a high induction from a gene expression from the non‐detectable level

To define the influence of GlaR on the expression of the *gal–lac* operon genes in response to various sugars, using RT‐qPCR, we compared mRNA levels of individual genes in *L. lactis* IL1403 wild‐type and IL1403Δ*glaR* grown in C‐M17, G‐M17, Gal‐M17 or GalC‐M17. Cellobiose in GalC‐M17 allowed *L. lactis* IL1403Δ*glaR* to grow in the presence of galactose, as this mutant is incapable to use galactose as a carbon source. In the presence of galactose, the expression of most of the Leloir operon genes was significantly lower in *L. lactis* IL1403Δ*glaR*, whereas in cellobiose‐ or glucose‐supplemented media, they were expressed at a similar level in both the strains (Figure 3a). The most pronounced difference between the strains concerned *lacS*, whose mRNA was virtually undetectable in *L. lactis* IL1403Δ*glaR* grown in the presence of any sugar tested, but was abundant in *L. lactis* IL1403 grown in galactose‐containing media (Gal‐M17 and GalC‐M17) (Figure 3a). Notably, *lacS* was not expressed in wild‐type *L. lactis* IL1403 grown without galactose. Some GlaR‐dependent activation in the presence of galactose was also observed for other *gal–lac* operon genes. The GlaR activation coefficient (calculated as the ratio of gene expression level in IL1403 to that in IL1403Δ*glaR*) for those genes varied between 2.6 (*galE*) and 8 (*galM*) (Figure 3b). For the negative control *yufC*, the GlaR activation coefficient was close to 1, indicating—as expected—a lack of GlaR‐dependent activation (Figure 3b).

The lowest transcript levels of the genes studied were detected in *L. lactis* IL1403 wild‐type and IL1403Δ*glaR* growing under repressive conditions (G‐M17), most likely due to the downregulation of *gal–lac* genes by CcpA, as described previously (Luesink et al., 1998). Expression of most of the Leloir genes increased in both strains in the medium supplemented with cellobiose (Figure 3a) indicating a release from catabolic repression. Notably, *lacS* mRNA was not detected in either of these media in either of the strains. In comparison with cellobiose, higher transcript levels of the *gal–lac* operon genes were detected when the wild‐type strain was grown in media supplemented with galactose (Gal‐M17 or GalC‐M17) (Figure 3a). The activation by galactose calculated as the ratio of expression in GalC‐M17 and in C‐M17 was the highest for the *lacS* gene, and for the other *gal–lac* genes, it varied from 1.6 (*galE*) to 6 (*galM*) (Figure 3b). In the *glaR* mutant downstream of *lacS,* these ratios were ca. 1 indicating that in the absence of GlaR, the galactose‐dependent activation of the *gal–lac* genes does not occur. Also for the negative control gene *yufC,* its expression levels with and without galactose were similar in both the wild‐type strain and in the *glaR* mutant further confirming that it is not subject to galactose induction (Figure 3B).

### 3.5 GlaR activates expression of the Leloir operon by binding to the *lacS* promoter region

**Figure 4:**
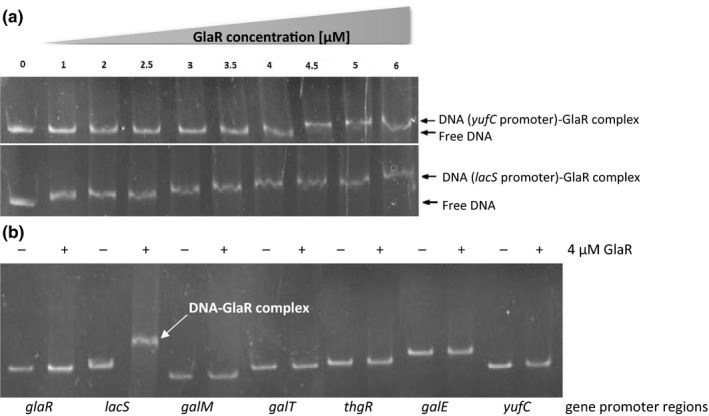
GlaR binding to potential promoter regions of *gal–lac* operon genes and *glaR*. The test was performed by electrophoretic mobility‐shift assays (EMSA) of (A) the GlaR protein gradient and *lacS* or *yufC* (negative control) ca. 200 nt putative promoter regions (B) and GlaR at concentration of 4 μM and ca. 200 nt putative promoter regions of selected genes of the Leloir operon plus *yufC*. “Free DNA” indicates DNA without bound GlaR; “DNA‐complex” indicates DNA with bound GlaR

To identify the genomic region to which the GlaR protein binds specifically, an in vitro EMSA test was performed with selected upstream regions containing potential promoters of the Leloir operon genes (*lacS*,* galM*,* galT*,* thgA,* and *galE*) and of *glaR* and purified GlaR protein. An unrelated dsDNA containing the *yufA* upstream region was used as a control to test for nonspecific binding. No nonspecific interactions were detected at GlaR concentrations up to 4 μM; therefore, this concentration was used to investigate specific binding (Figure 4a). At this concentration, GlaR bound to the putative *lacS* promoter but it did not form specific complexes with any other putative promoters tested (Figure 4b). Notably, GlaR bound to the *lacS* dsDNA also at lower concentrations (1–3.5 μM) (Figure 4a), indicating that the interaction is fairly strong.

### 3.6 *GlaR* expression is inducible by galactose but insensitive to CcpA‐mediated catabolite repression

CcpA is a master transcriptional regulator controlling carbohydrate utilization and metabolism genes in gram‐positive bacteria including *L. lactis* (Hueck & Hillen, 1995; Zomer et al., 2007). As the promoter region of *glaR* contains a potential *cre* sequence (Figure 1) that could be recognized by CcpA, we sought to determine the role of CcpA in the transcriptional regulation of this gene. *glaR* expression in the presence of different sugars (glucose, galactose, or cellobiose) was compared between the wild‐type strain and a *ccpA* mutant (IL1403*ccpA* ^‐^). The lack of the CcpA regulatory protein had no effect on *glaR* expression in any of the media tested, indicating that *glaR* is not under CcpA‐dependent catabolite repression. On the other hand, the transcription of *glaR* in IL1403 was elevated ca. sevenfold in galactose‐containing media compared to its expression in glucose‐ or cellobiose‐supplemented media (Figure 3b), and indicating a possible autoregulation of the *glaR* gene.

## 4 DISCUSSION

Because of the substantial biotechnological relevance of galactose, especially in the dairy industry, where unmetabolized galactose is associated with poor product quality (Baskaran & Sivakumar, 2003; Hutkins, Halambeck, & Morris, 1986; Michel & Martley, 2001), galactose metabolism and its regulation have been thoroughly studied in several LAB species. The crucial role of the Leloir pathway in the utilization of nonphosphorylated galactose is well documented. In several species of the genera *Lactobacillus* and *Streptococcus*, the Leloir or *gal–lac* operons are known to be regulated transcriptionally mainly by repressor proteins belonging to the LacI family, but the regulatory mechanism of the Leloir pathway genes in *L. lactis*, important dairy industry bacterium, remained unexplored to date. In this study, we demonstrate that in *L. lactis,* the regulation of the *gal*–*lac* operon differs from that in other LAB species as it is under a positive control of the RpiR‐family transcriptional regulator GlaR.

The *L. lactis* IL1403 genome carries eight genes encoding RpiR‐family members: GlaR (previously named YugA), ClaR (previously YebF), GntR, YecA, YfeA, YidA, YljC, and YleF (retrieved from http://www.kegg.jp/kegg/ssdb/). Thus far, only one RpiR‐member, ClaR, has been characterized in *L. lactis* and shown to function as an activator of cellobiose and lactose metabolism genes *bglS* and *celB* (Aleksandrzak‐Piekarczyk et al., 2015). In other species distantly related to *L. lactis*, members of the RpiR family have been found to function as regulators targeting genes involved in the metabolism of diverse carbon sources. Thus, GlvR is a positive regulator of maltose metabolism in *B. subtilis* (Yamamoto, Serizawa, Thompson, & Sekiguchi, 2001), HexR, IolR, MurR, and RpiR act as repressors of glucose, inositol, N‐acetylmuramic acid, ribose or central carbon metabolism in several gram‐negative bacteria (Antunes et al., 2016; Jaeger & Mayer, 2008; Kohler, Choong, & Rossbach, 2011; Sørensen & Hove‐Jensen, 1996), and HexR is a dual‐mode pleiotropic regulator of the central carbohydrate metabolism in proteobacteria (Leyn et al., 2011). Thus far, none of the RpiR regulators has been implicated in modulating galactose metabolism.

Members of the RpiR family harbor a DNA‐binding HTH domain and a phospho‐sugar‐binding SIS motif, respectively, at their N‐ and C‐terminal regions (Bateman, 1999; Teplyakov, Obmolova, Badet‐Denisot, Badet, & Polikarpov, 1998). The SIS domain is found in numerous proteins that regulate expression of genes dedicated to the synthesis of phospho‐sugars (Aleksandrzak‐Piekarczyk et al., 2015; Bateman, 1999; Daddaoua, Krell, & Ramos, 2009; Jaeger & Mayer, 2008; Sørensen & Hove‐Jensen, 1996; Teplyakov et al., 1998; Yamamoto et al., 2001) but here, we show that a protein from this family can also be engaged in regulating of an operon involved in the metabolism of a nonphosphorylated sugar galactose. This mode of regulation seems to be restricted to the genus *Lactococcus* as well‐conserved GlaR homologues occur only in these bacteria and their genes are always localized directly upstream of the *gal–lac* or Leloir operons.

Using two *L. lactis* strains differing by the presence of *glaR* and growing them in media with different sugars as the sole carbon source, we showed that the *gal–lac* operon genes are maximally expressed only when both galactose and GlaR are available. This effect was absolute for the *lacS* gene, as its transcript was virtually undetectable in the absence of galactose or GalR. For the other genes located downstream of *lacS,* the GlaR‐dependent induction by galactose was less spectacular and, notably, its extent decreased with increasing distance from *lacS*. These results suggested that the genes in question form a single operon with the promoter preceding the *lacS* gene. Indeed, using EMSA, we found that GlaR does bind specifically to a region upstream of *lacS*, but not to the putative promoters of the other genes downstream of *lacS*. Notably, the presence of GlaR‐independent promoter‐like regions upstream of these genes explains why they were expressed at a submaximal level even in the absence of galactose/or GlaR. We additionally confirmed that functional expression of *lacS* requires the action of GlaR by showing that the *L. lactis* IL1403Δ*glaR* was unable to grow in galactose medium. LacS permease is the main transporter used for galactose (but not for lactose; Aleksandrzak‐Piekarczyk et al., 2005) uptake in IL1403 cells, and its inactivation leads to the *gal* ^‐^ phenotype (our unpublished data).

Remarkably, also the transcription of *glaR* was induced substantially in galactose‐containing medium in comparison with cellobiose, which could in part explain the effect of galactose on the GlaR‐dependent expression of the *gal–lac* operon. It also suggested possible autoregulation of *glaR* expression by GlaR. Autoregulation is frequent in prokaryotic gene regulation strategies and has been reported for numerous transcription regulators (Gerlach, Valentin‐Hansen, & Bremer, 1990; Meng, Kilstrup, & Nygaard, 1990; Morel, Lamarque, Bissardon, Atlan, & Galinier, 2001; Vaughan et al., 2001; Weickert & Adhya, 1993). However, we could not confirm a direct involvement of GlaR in *glaR* activation as no GlaR binding to the *glaR* promoter region was found by EMSA (Figure 4b). A plausible explanation includes an indirect control by GlaR (e.g., via an alternative regulator under the control of GlaR) or the action of another galactose‐dependent but GlaR‐independent mechanism.

Both *lacS* and *glaR* are preceded by *cre* boxes suggesting that their expression is under CcpA‐driven carbon catabolite repression (CCR). Indeed, in the presence of glucose, transcriptional arrest of all the genes under the control of the *lacS* promoter was detected, whereas cellobiose or galactose caused a relief from CCR. This phenomenon has already been studied in another *L. lactis* strain, MG1363 (Luesink et al., 1998), in which the Leloir operon differs from the one of IL1403 but is also subject to CcpA‐driven catabolic repression. In contrast, we found that that CcpA is not engaged in the regulation of *glaR* expression in *L. lactis* IL1403. One reason for this could be the two‐nucleotide deviation of the *cre* sequence upstream of *glaR* (TaAAAACGaTTTCA) form the *cre* consensus WGWAARCGYTWWMA (Zomer et al., 2007). The two adenine mismatches may prevent or impair CcpA interaction with its operator and thus allow of the *glaR* transcription also in repressive conditions (glucose).

In summary, here, we have documented unusual mechanism of *gal–lac* operon activation in *L. lactis* IL1403 and, by similarity, probably also in other *Lactococcus* spp. No similar mechanism has been reported in other LAB species. This regulation relies on galactose‐inducible and GlaR‐dependent transcriptional activation of the *lacS* promoter inducing the *lacS* gene itself and the other *lac* and Leloir pathway genes located downstream.

## ETHICAL STATEMENT

This article does not contain any studies with human or animals performed by any of the authors.

## CONFLICT OF INTEREST

The authors declare that they have no conflict of interest.

## Data Availability

The authors declare that all data generated or analyzed during this study are included in this article.
